# Dendritic cell dysfunction, including impaired IL-12 production, is associated with chronic pulmonary aspergillosis

**DOI:** 10.1093/cei/uxaf038

**Published:** 2025-06-12

**Authors:** Stefano A P Colombo, Sara Gago, Mathilde Chamula, Robert Lord, Andrew S MacDonald, Chris Kosmidis

**Affiliations:** Lydia Becker Institute of Immunology and Inflammation, Faculty of Biology, Medicine, and Health, Manchester Academic Health Science Centre, The University of Manchester, Manchester, UK; Manchester Fungal Infection Group, Faculty of Biology, Medicine, and Health, Manchester Academic Health Science Centre, University of Manchester, Manchester, UK; National Aspergillosis Centre, Department of Infectious Diseases, Manchester University NHS Foundation Trust, Manchester, UK; Lydia Becker Institute of Immunology and Inflammation, Faculty of Biology, Medicine, and Health, Manchester Academic Health Science Centre, The University of Manchester, Manchester, UK; Department of Respiratory Medicine, Manchester University NHS Foundation Trust, Manchester, UK; Manchester Adult Cystic Fibrosis Centre, Manchester University NHS Foundation Trust, Manchester, UK; Lydia Becker Institute of Immunology and Inflammation, Faculty of Biology, Medicine, and Health, Manchester Academic Health Science Centre, The University of Manchester, Manchester, UK; Manchester Fungal Infection Group, Faculty of Biology, Medicine, and Health, Manchester Academic Health Science Centre, University of Manchester, Manchester, UK; National Aspergillosis Centre, Department of Infectious Diseases, Manchester University NHS Foundation Trust, Manchester, UK

**Keywords:** fungal, dendritic cells, cellular immunology, cytokines

## Abstract

**Background:**

Growing evidence links immune dysfunction, notably impaired IFNγ production, to chronic pulmonary aspergillosis (CPA), but understanding of the immune phenotype in CPA patients remains limited.

**Methods:**

To investigate this, we recruited 25 CPA patients and 25 controls with bronchiectasis, isolating immune cells from peripheral blood for detailed flow cytometric phenotyping at resting state and after *ex vivo* stimulation with the TLR2/Dectin-1 agonist Zymosan.

**Results:**

CPA patients exhibited pronounced neutrophilia and a reduced frequency of conventional dendritic cell (DC) subsets at baseline compared to bronchiectasis controls. Post-stimulation, DC and monocyte subsets in CPA patients showed significantly lower expression of activation markers. Notably, cDC1s displayed reduced IL-12p40, TNFα, and CD86 expression. CPA patients with a history of tuberculosis (TB) had significantly higher frequencies of activated cDC1s. Machine learning analysis validated these immunological parameters as predictive of CPA status.

**Conclusion:**

Our findings suggest that immune dysfunction in CPA involves DC and monocyte impairments, potentially contributing to IFNγ deficiency through reduced IL-12 production and co-stimulatory capacity in cDC1s. These results also hint at the presence of innate immune memory in CPA patients with prior TB. Our study advances understanding of the immune dysfunction underlying CPA.

## Teaser

Growing evidence links immune dysfunction, notably impaired IFNγ production, to chronic pulmonary aspergillosis (CPA), but an understanding of the immune phenotype in CPA patients remains limited. To investigate this, we recruited 25 CPA patients and 25 controls with bronchiectasis, isolating immune cells from peripheral blood for detailed flow cytometric phenotyping at resting state and after ex vivo stimulation with the TLR2/Dectin-1 agonist Zymosan. Our findings suggest that immune dysfunction in CPA involves DC and monocyte impairments, potentially contributing to IFNγ Deficiency through reduced IL-12 production and co-stimulatory capacity in cDC1s.

## Graphical Abstract

**Graphical Abstract:**
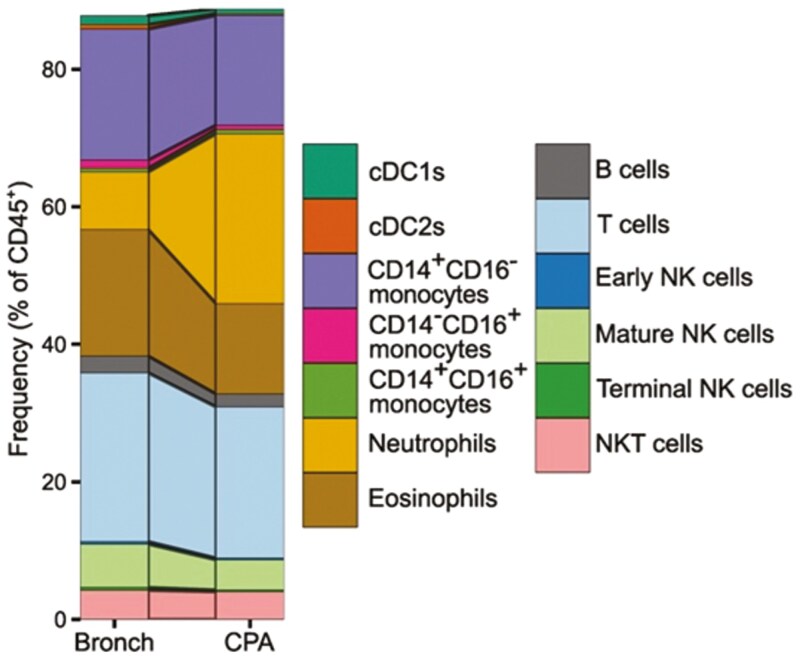

## Introduction

Pulmonary infections caused by *Aspergillus* species represent a significant global health challenge, with the potential to become life-threatening for afflicted individuals [1]. Chronic pulmonary aspergillosis (CPA) is a progressive infection known to opportunistically exploit pre-existing pulmonary conditions, with a notable prevalence of prior tuberculosis (TB) infection and chronic obstructive pulmonary disease (COPD) among these patients [2]. While the primary treatment for CPA involves the administration of azole-class drugs, post-treatment relapse is a common occurrence, often accompanied by the development of azole resistance [1, 2]. These compounding factors contribute to poor long-term prognosis for patients [2], emphasizing the urgent need to gain insight into the factors that render individuals susceptible to disease.

CPA typically occurs in patients thought to be immunocompetent [2]. However, we recently identified a deficiency in interferon-gamma (IFNγ) production in a cohort of CPA patients [3]. Strikingly, the level of IFNγ production in whole blood culture, in response to *ex vivo* stimulation with fungal pathogen-associated molecular patterns (PAMPs), was predictive of long-term survival [3], highlighting the key role played by the immune system in CPA. However, at present, there is limited understanding of the immune phenotype of CPA patients. Studies on cytokine levels measured in the serum and bronchoalveolar lavage fluid (BALF) of CPA patients have identified a common signature of elevated IL-1β, IL-6, and IL-8 relative to healthy controls [4–6]. Additionally, monocyte-derived macrophages generated from CPA patients express higher levels of IL1A, IL1B, and IL6 transcript compared to those generated from healthy controls when exposed to live *Aspergillus fumigatus* conidia *ex vivo* [7]. Interestingly, we have previously observed that CPA can impair responsiveness to vaccination against pneumococcal polysaccharides [8], supporting the importance of immunity during CPA. Increasing depth of understanding at the cellular level in this understudied area is essential in order to leverage host immunity to support therapeutic intervention in CPA.

Murine models of aspergillosis, and other pulmonary fungal infections, centre the role for interleukin (IL)-17A and T helper 17 (Th17) cells as key mediators of anti-fungal immunity [9]. Additionally, a growing role for dendritic cells (DCs), particularly plasmacytoid DCs (pDCs) [10, 11] and conventional type-1 DCs (cDC1s) [12], has been reported. However, the extent to which these observations translate to human aspergilloses is unclear. This is especially true of CPA, the key feature of which is long-term chronic disease which has not yet been accurately replicated in murine systems.

cDC1s are a subset of cDCs specialized for the induction of the cytotoxic activity of CD8^+^ T cells and polarization of Th cells to a Th1 phenotype [13]. This activity is mediated in part through the production of IL-12 [13]. In humans, a range of cell surface markers can distinguish cDC1s from type-2 cDCs (cDC2s), notably, cDC1s do not express the canonical cDC2 markers CD11b and CD1c [13]. Whilst the role of specific DC subsets has not been investigated in the context of CPA, studies on *ex vivo* cultured human monocyte-derived DCs indicate that, in response to exposure to *A. fumigatus,* they upregulate co-stimulatory molecules and produce type-1 cytokines including IL-12 and TNFα [14–16]. This suggests that human DCs are primed for maturation into a pro-inflammatory phenotype in response to *Aspergillus* exposure.

Addressing the need for deeper understanding of the immunology of CPA, we present the first high-parameter flow cytometric immune phenotyping of peripheral immune cells isolated from CPA patients. We show that CPA patients retain an intact peripheral lymphocyte compartment but have reduced frequencies of cDC and monocyte subsets, relative to controls. We also provide evidence that myeloid cells isolated from CPA patients, particularly cDC1s, are impaired in their ability to upregulate maturation markers and type-1 cytokines following *ex vivo* stimulation. Finally, we apply a machine-learning approach to demonstrate that immunological parameters can be used to predict poor outcomes in CPA, highlighting the diagnostic potential of targeted immune phenotyping.

## Results

### The peripheral blood of CPA patients is characterized by pronounced neutrophilia and decreased cDC frequency.

To gain insight into the immune phenotype of CPA patients, we isolated immune cells from the peripheral blood of CPA donors and non-CPA bronchiectasis controls (Table 1). As CPA is typically comorbid with other chronic inflammatory pulmonary conditions we chose patients with bronchiectasis to allow identification of immune features of CPA specific to CPA, as opposed to features associated more broadly with airway inflammation. Although patients with bronchiectasis have a marked neutrophilic inflammatory background [17] and are susceptible to chronic colonization of the airways by microbes including *Aspergillus* [18], not all patients with bronchiectasis will also develop CPA in parallel. Within our CPA cohort bronchiectasis was the most frequent comorbidity (65.2%, Table 1), making non-CPA bronchiectasis patients ideal controls. For all donors, leukocytes were isolated by red blood cell (RBC) lysis and, for deeper phenotyping of myeloid cells and T cells, enriched for peripheral blood mononuclear cells (PBMCs) by Ficoll-Paque density centrifugation.

**Table 1:** summary of donor cohort demographics and clinical Features

	Bronchiectasis	CPA
**Age** (y, median(range))	69 (28–78)	69 (35–77)
**Sex** (F, (count (%))	14 (60.9)	6 (26.1)
**Bronchiectasis** (count (%))	23 (100)	15 (65.2)
**Asthma** (count (%))	12 (52.2)	3 (13)
**COPD** (count (%))	2 (8.7)	13 (56.5)
**Diabetes** (count (%))	4 (17.4)	3 (13)
**Smoking history** (count (%))		
Never smoker	17 (73.9)	5 (21.7)
Ex-smoker	4 (17.4)	12 (52.2)
Current smoker	1 (4.3)	4 (17.4)
Not recorded	1 (4.3)	2 (8.7)
**CPA clinical features**		
**Aspergilloma** (count (%))	–	15 (65.2)
**Bilateral disease** (count (%))	–	2 (8.7)
**Prior TB** (count (%))	2 (8.7)	7 (30.4)
**Prior NTM** (count (%))	–	1 (4.3)

**Figure 1:**
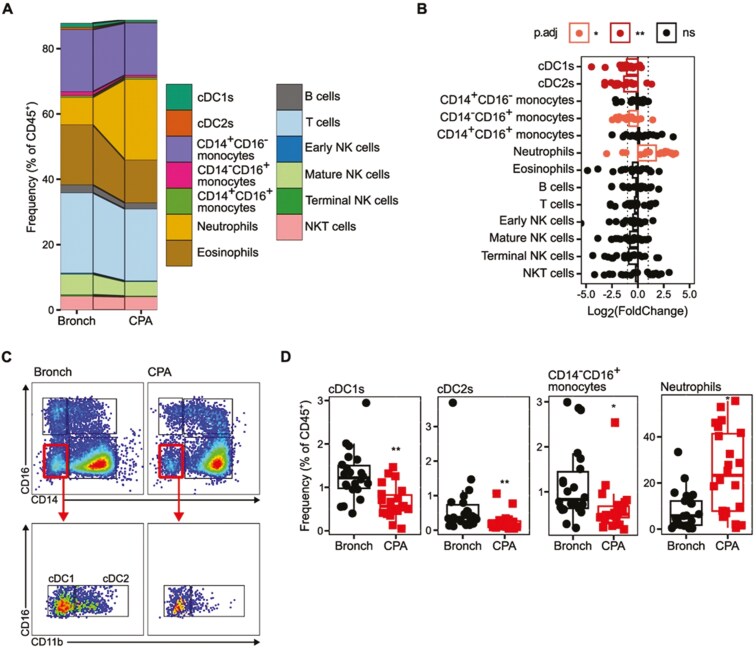
CPA is associated with neutrophilia and reduced frequency of classical dendritic cells in peripheral circulation. Whole blood was collected from donors with CPA and control bronchiectasis (Bronch) donors. Red blood cells were lysed and immune cells were phenotyped by flow cytometry. CPA *n* = 22, Bronch *n* = 22. (**A**) Stacked bar plot representing the mean frequency of immune cell subsets as a proportion of live CD45^+^ cells. (**B**) Scatter plot indicating the log_2_(fold change) for the frequency of immune cells subsets in CPA donors relative to the geometric mean of the Bronch group. Individual point represent individual donors, bars indicate the mean log_2_(fold change) of the CPA group, dotted lines indicate -1 and 1, colour denotes the adjusted *P* value (*P*.adj) for statistical comparison between the CPA group and Bronch group as calculated by unpaired Wilcoxon test with correction using the Holm correction method. (**C**) Representative flow cytometry plots for the gating of monocyte and cDC subsets. Representative plots are from a combination of five random CPA donors and five random Bronch donors. (**D**) Boxplots representing the raw data for the frequency of cDC1s, cDC2s, CD14^−^CD16^+^ monocytes, and neutrophils as a proportion of live CD45^+^ cells in Bronch and CPA donors. Boxplots indicate the median and interquartile ranges. Whiskers indicate the minimum/maximum value within 1.5× the lower/upper quartile limit. Points indicate individual donors. *P.adj < 0.05, **P.adj < 0.01

We first assessed the overall composition of the circulating immune cell population, in samples isolated by RBC lysis, by flow cytometry (Supplementary Fig. S1A, Fig. 1A). Processing and storage conditions resulted in a low recovery of granulocytes, resulting in a lower than typical frequency of neutrophils in both cohorts (Fig. 1A). We observed marked differences between CPA and bronchiectasis controls for a range of key immune subsets (Fig. 1B). Notably CPA patients exhibited a significant increase in the frequency of neutrophils as a proportion of total CD45^+^ cells relative to bronchiectasis controls (Fig. 1D). Interestingly, we observed significant changes in frequency of cell subsets in the mononuclear phagocyte (MNP) compartment, defined as CD11c^+^HLA-DR^+^ cells (Fig. 1C). Both cDC1s (CD14^-^CD16^-^CD11b^-^) and cDC2s (CD14^−^CD16^−^CD11b^+^) were significantly reduced in frequency in CPA patients compared to controls (Fig. 1D). Additionally, non-classical CD14^-^CD16^+^ monocytes were reduced in frequency in CPA patients (Fig. 1D). However, classical CD14^+^CD16^-^ monocytes and intermediate CD14^+^CD16^+^ monocytes were found at the same frequency in CPA patients as controls (Fig. 1B). No significant difference in the frequency of total T cells, B cells or NK cell subsets, as a proportion of total circulating CD45^+^ cells, was observed (Fig. 1B).

### T-cell memory phenotype is maintained during CPA.

**Figure 2:**
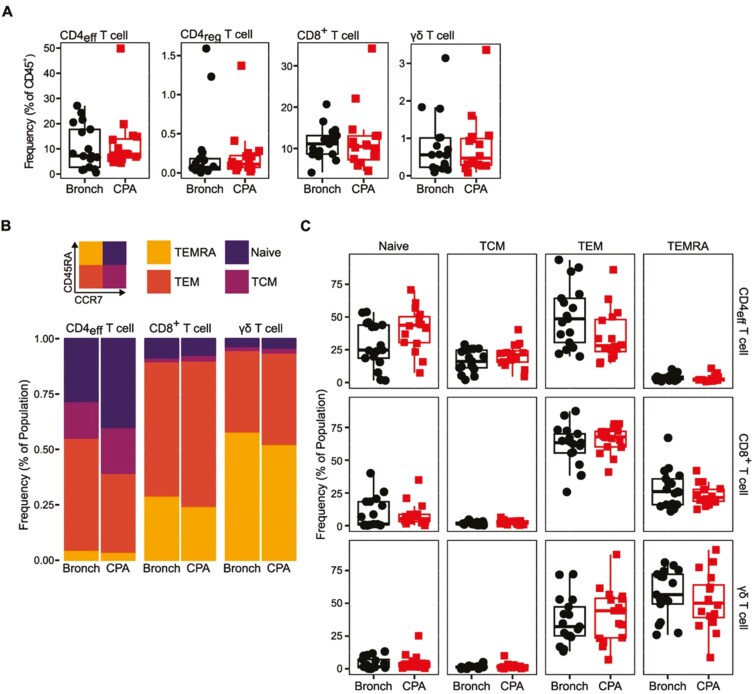
memory phenotype is maintained in peripheral circulating T cells during CPA. Peripheral blood mononuclear cells (PBMCs) were isolated from CPA and control Bronchiectasis (Bronch) donors, and then phenotyped by flow cytometry. CPA *n* = 16, Bronch *n* = 17. (**A**) Boxplots representing the frequency of T-cell subsets in CPA and Bronch donors as a frequency of live CD45^+^ PBMCs. Effector CD4 T cells (CD4_eff_) defined as CD3^+^CD4^+^CD25^-^. Regulatory CD4 T cells (CD4_reg_) defined as CD3^+^CD4^+^CD25^+^CD127^-^. CD8 T cells are defined as CD3^+^CD8^+^. γδ T cells are defined as CD3^+^TCRγδ^+^. (**B**) Stacked bar plots indicating the memory phenotype of T-cell subsets in Bronch and CPA donors. Memory phenotype was determined using expression of CD45RA and CCR7 giving four populations; Naïve (CD45RA^+^CCR7^+^), T central memory (TCM, CD45RA^-^CCR7^+^), T effector memory (TEM, CD45RA^−^CCR7^−-^), and T effector memory expressing CD45RA (TEMRA, CD45RA^+^CCR7^-^). Bars indicate the relative abundance of a given memory phenotype as a frequency of that T-cell subsets population. (**C**) Boxplots representing the raw data for the frequency of memory phenotypes in T-cell subsets. Boxplots indicate the median and interquartile ranges. Whiskers indicate the minimum/maximum value within 1.5× the lower/upper quartile limit. Points indicate individual donors

We next assessed PBMC T-cell populations in greater depth using T-cell subset defining markers and flow cytometry (Fig. 2). We defined four major T-cell populations based on surface marker expression: effector CD4 T cells (CD4_eff_, CD3^+^TCRγδ^-^CD4^+^CD8^-^CD25^−^), regulatory CD4 T cells (CD4_reg_, CD3^+^TCRγδ^−^CD4^+^CD25^+^CD127^−^), CD8 T cells (CD3^+^TCRγδ^−^CD4^-^CD8^+^), and γδ T cells (CD3^+^TCRγδ^+^) (Supplementary Fig. S1B). All four T-cell subsets were found at comparable frequencies in CPA patients when compared to bronchiectasis controls (Fig. 2A). We also measured the memory phenotype of CD4_eff_, CD8 and γδ T cells using expression of CCR7 and CD45RA to define four memory states: Naïve T cells (CCR7^+^CD45RA^+^), central memory T cells (TCM, CCR7^+^CD45RA^-^), effector memory T cells (TEM, CCR7^−^CD45RA^−^), and effector memory T cells expressing CD45RA (TEMRA, CCR7^−^CD45RA^+^) [19] (Fig. 2B). We did not identify a statistically significant difference in the frequency of any T-cell memory phenotype in CPA patients, compared to bronchiectasis controls (Fig. 2C).

### Maturation potential is impaired in cDC1s isolated from peripheral blood of CPA patients.

Given our previous data demonstrating reduced cytokine potential in CPA patients in response to fungal PAMPs, as assessed in the supernatants of *ex vivo* whole blood cultures [3], and the reduced frequency of MNP subsets identified here, we next asked whether the ability of MNPs to respond to fungal PAMPs was impaired in our CPA cohort. To do this we cultured PBMCs isolated from CPA patients and bronchiectasis controls overnight in the presence of ZYM (10 μg/ml) and then assessed the MNP activation state by flow cytometry (Supplementary Fig. S2).

**Figure 3:**
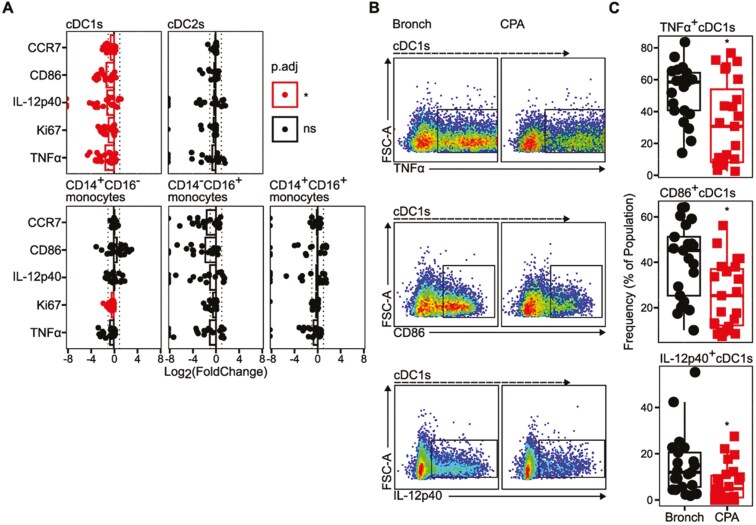
CPA is associated with reduced activation potential in cDC1s following ex vivo stimulation. Peripheral blood mononuclear cells (PBMCs) were isolated from control bronchiectasis (Bronch) and CPA donors. Cells were cultured for 16 h in the presence of Zymosan (ZYM, 10 μg/ml). Cells were phenotyped by flow cytometry. CPA *n* = 23, Bronch *n* = 21. (**A**) Scatter plots indicating the log_2_(fold change) of the frequency of marker positive cells, as a proportion of a given cell type, in CPA donors relative to the median of the Bronch group. Points represent individual donors, bars indicate the mean log_2_(fold change) of the CPA group, dotted lines indicate -1 and 1, colour denotes the adjusted *P* value (*P*.adj) for statistical comparison between the CPA group and Bronch group as calculated by unpaired Wilcoxon test with correction using the Holm correction method. (**B**) Representative flow cytometry plots for the expression of TNFα, CD86, and IL-12p40 in cDC1s in control and CPA donors. Representative plots are from a combination of five random CPA donors and five random control donors. (**C**) Boxplots indicating the expression of TNFα, CD86, and IL-12p40 by cDC1s are given as the frequency of marker-positive cells as a proportion of total cDC1s. Boxplots indicate the median and interquartile ranges. Whiskers indicate the minimum/maximum value within 1.5× the lower/upper quartile limit. Points indicate individual donors. *P.adj < 0.05

We noted a significant decrease in the frequency of cDC1s (CD14^-^CD16^-^CD1c^-^) and CD14^+^CD16^−^ monocytes expressing a range of activation markers including CCR7, IL-12p40, CD86, Ki-67, and TNFα, as a proportion of total CD45^+^ PBMCs in CPA patients compared to bronchiectasis controls following ZYM stimulation (Supplementary Fig. S3A). Further, we identified a specific reduction of expression of these activation markers by cDC1s from these patients when assessing expression as a frequency of the total cDC1 population (Fig. 3A-C). We also noted a statistically significant reduction in the frequency of Ki-67^+^ CD14^+^CD16^-^ monocytes as a proportion of total CD14^+^CD16^-^ monocytes (Fig. 3A).

### Prior diagnosis with TB is associated with higher frequency of activated cDC1s following stimulation.

Immune phenotype can be influenced by a range of environmental and clinical covariates. To understand whether other factors were contributing to the composition of the circulating immune cell population in our CPA cohort, we compared the immune profile of patients within the CPA group based on a range of variables. Sex and smoking history showed no significant impact on the frequency of major immune cell populations as a proportion of total circulating CD45^+^ cells isolated by RBC lysis, nor did the presence of an aspergilloma on CT scan or whether the infection was bilateral (Supplementary Fig. S3B). However, CPA patients with a history of asthma had a significantly higher frequency of eosinophils present in their blood and a concomitant decrease in the frequency of NKT cells compared to non-asthmatic CPA patients (Supplementary Fig. S3C). Additionally, CPA patients diagnosed with COPD had a significantly reduced frequency of cDC1s (Supplementary Fig. S3C) and those with diabetes had a significant increase in the frequency of CD14^+^CD16^-^ monocytes (Supplementary Fig. S3C).

**Figure 4:**
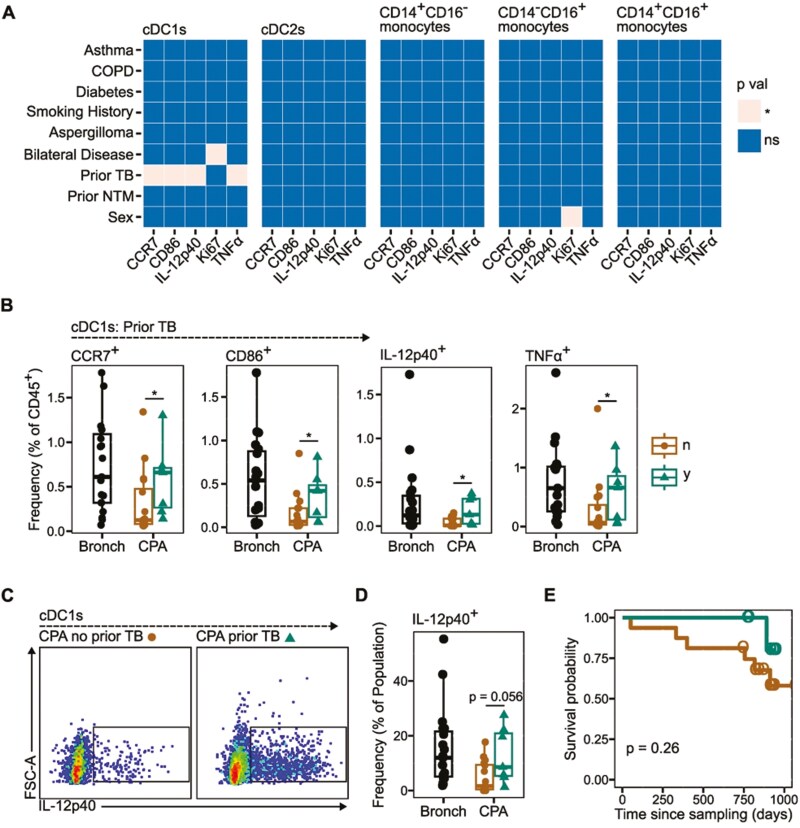
prior diagnosis with Tuberculosis (TB) correlates with higher frequency of activated cDC1s following *ex vivo* stimulation. (**A**) Tile plot indicating the results of statistical comparisons for CPA donors based on clinical covariates, in which the expression of mononuclear phagocyte (MNP) activation markers (given as marker positive cells as a frequency of live CD45^+^ cells) on different MNP cell types is compared between donors with that covariate (*y*) compared to those without (*n*), e.g. CPA donors with Asthma (*y*) compared to those without asthma (*n*). Each tile indicates a statistical comparison. Tile colour indicates the calculated *P* value. (**B**) Boxplots indicating the frequency of cDC1s expressing activation markers were determined to be statistically significant given the frequency of live CD45^+^ cells for CPA donors stratified based on previous TB diagnosis (*y* = prior TB diagnosis, *n* = no prior TB diagnosis). Bronch *n* = 19, CPA-n *n* = 16, CPA-y *n* = 7. (**C**) Representative flow cytometry plots for the expression of IL-12p40 by cDC1s in CPA donors stratified by prior TB diagnosis. Representative plots are from a combination of five random CPA donors with a prior TB diagnosis and five without. (**D**) Boxplot indicating the frequency of IL-12p40 expression by cDC1s given as frequency of the total cDC1 population in CPA donors stratified by prior TB diagnosis and in Bronchiectasis donors (Bronch). Boxplots indicate the median and interquartile ranges. Whiskers indicate the minimum/maximum value within 1.5x the lower/upper quartile limit. Points indicate individual donors. *P* values were calculated by unpaired Wilcoxon test. *P < 0.05. (**E**) Kaplan–Meier survival analysis. Lines show Kaplan–Meier curves for CPA patients without prior TB (brown) and with prior TB (green). Circles denote points of censure. *P* value was calculated by log-rank test

When we compared the frequency of activation maker positive MNPs within the CPA group, based on clinical covariates, there was a clear signature for increased frequency of activation marker positive cDC1s in CPA patients with a prior TB diagnosis, following ZYM stimulation, as a proportion of CD45^+^ PBMCs (Fig. 4A). We identified a significantly higher frequency of cDC1s expressing CCR7, CD86, IL-12p40, and TNFα (Fig. 4B) as a proportion of total CD45^+^ PBMCs in CPA patients with a history of TB. Additionally, there was a strong trend for increased IL-12p40 expression in cDC1s from CPA patients with a history of TB as a frequency of total cDC1s (Fig. 4C), although this did not reach statistical significance (Fig. 4D). A Kaplan–Meier survival analysis identified a trend for higher survival probability in patients with a history of TB, however, this was not statistically significant (Fig. 4E). Given that, in our patient cohort, CPA patients with a prior TB diagnosis were on average 10 years younger than those without a history of TB (Supplementary Fig. S4A), we assessed whether age was a driving factor in the differential potential for cDC1 maturation in CPA patients with prior TB. Interestingly we observed trends for positive correlations between age and the frequency of CCR7^+^, CD86^+^, IL-12p40^+^, and TNFα^+^ cDC1s in CPA patients with a history of TB, but the reverse was true for those patients without prior TB infection (Supplementary Fig. S4B). However, for the most part, these correlations did not reach statistical significance.

### A random forests machine learning analysis identifies a population of CPA patients with poor survival outcome

**Figure 5:**
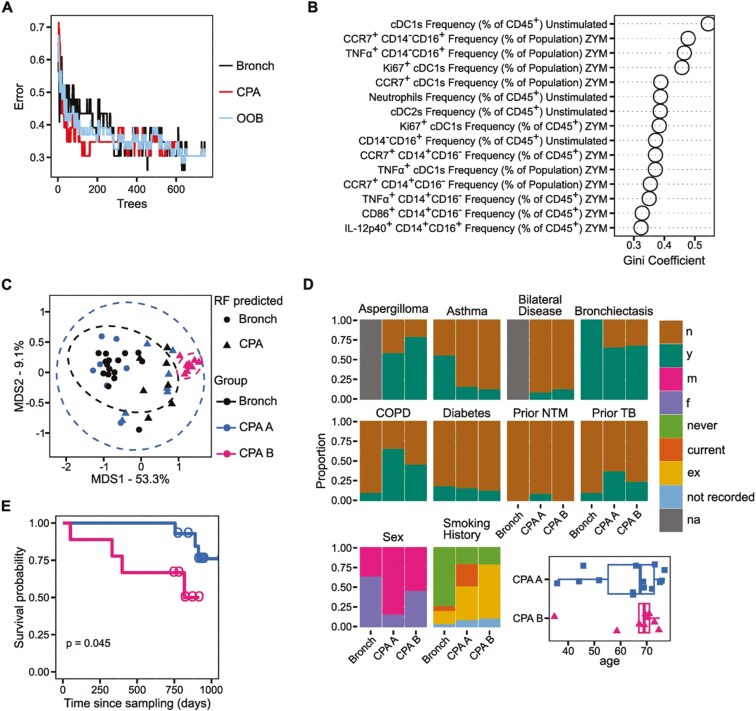
immune phenotyping predicts CPA patients with poor survival outcomes in a Random Forest analysis. Random forest analysis was performed using the randomForest package in R using all recorded immune parameters (mtry = 3, ntree = 750). (**A**) Line graph representing the out-of-bag error rate of the random forest (RF) model with increasing number of trees. (**B**) Top 15 parameters ranked by Gini coefficient calculated by the RF model. Higher Gini coefficient indicates higher importance in the RF model. (**C**) Classical multi-dimensional scaling (MDS) ordination plot calculated from sample proximity within the RF model. Shape indicates the group each patient was assigned to by the RF model (circle indicates bronchiectasis (Bronch) control, triangle indicates CPA). Colour indicates the group to which the patient belongs to (black indicates Bronch, blue and pink indicate CPA). CPA patients were manually stratified into two groups CPA A (blue) and CPA B (pink) based on clustering in the MDS analysis. Bronch *n* = 23, CPA A *n* = 14, CPA B *n* = 9. (**D**) Stacked bars representing the frequency of clinical covariates in control, CPA A, and CPA B patients. *n* = no, y = yes, m = male, f = female, never = never smoker, current = current smoker, ex = ex-smoker, not recorded = smoking status not recorded, na = not applicable. (**E**) Kaplan–Meier survival analysis. Lines show Kaplan–Meier curves for CPA patients stratified into CPA A (blue) and CPA B (pink). Circles denote points of censure. *P* value was calculated by log-rank test

To gain deeper insight into the immune phenotype of CPA patients, we applied a random forests (RFs) machine learning approach to our dataset. RFs are a supervised classification method that utilises aggregated, randomly generated decision trees. Importantly, RFs can incorporate high-dimensional datasets, be used for sample clustering, and be interrogated to measure the relative importance of variables [20]. We built an RF model utilizing all of our flow cytometry readouts, but not including clinical covariates. Our RF model had an estimated error rate of 30.43%, indicating a better-than-chance ability of the model to discriminate between bronchiectasis control and CPA patients when relying solely on their immune profile (Fig. 5A). To identify the features in our dataset that were most important in classifying patients, we extracted the Gini coefficients for each of our parameters. The frequency of cDC1s had the highest Gini coefficient, indicating strong importance in our RF model (Fig. 5B). Additionally, the activation state of cDC1s and monocyte subsets following ZYM stimulation were among the highest-ranked features (Fig. 5B).

**Figure 6:**
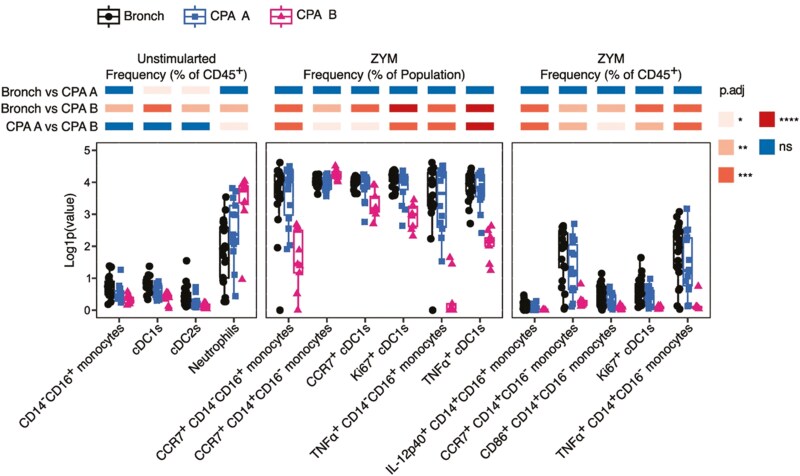
CPA patients with poor survival outcomes predicted by machine learning have an exaggerated immune phenotype. Boxplots representing the top 15 immune parameters ranked by Gini coefficient in a Random Forests (RF) machine learning model, with CPA patients stratified into CPA A and CPA B groups based on clustering within the model. Boxplots indicate the median and interquartile ranges. Whiskers indicate the minimum/maximum value within 1.5× the lower/upper quartile limit. Points indicate individual donors. Tiles above each parameter indicate the result of statistical comparisons between group. Colour indicates *P* value calculated by unpaired Wilcoxon test with correction using the Holm correction method. ns = not significant, *P.adj < 0.05, **P.adj < 0.01, *** *P*.adj < 0.001, **** *P*.adj < 0.0001.

We next used our RF model to cluster samples by first creating a distance matrix from sample proximity within the model, and then performing classical multidimensional scaling (MDS). MDS analysis identified a subset of CPA patients that were all accurately classified by the RF model and clustered tightly together (Fig. 5C). We labelled these patients ‘CPA B’ and remaining CPA patients ‘CPA A’ then compared clinical co-variates between the two groups to determine if there were non-immune features that correlated with this clustering. Notably, patients in the CPA B group had a higher frequency of aspergilloma seen on CT scans than those in the CPA A group (Fig. 5D). Strikingly, donors in the CPA B group had significantly poorer survival probability in a Kaplan–Meier analysis, compared to those in the CPA A group (Fig. 5E). Finally, we repeated the statistical analysis of the 15 immune parameters with the highest Gini coefficients in the RF model with the CPA A and CPA B groups separated. This revealed the CPA B group to have the lowest frequencies of cDC1s and cDC2s but the highest frequency of neutrophils (Fig. 6). Additionally, CPA B patients had the lowest expression of activation markers on cDC1s and, interestingly, this analysis identified these patients to have significantly reduced expression of activation markers on multiple monocyte subsets (Fig. 6).

## Discussion

Despite its considerable importance as a globally relevant inflammatory disease [2], there is little immunological understanding of CPA. There has been long-standing clinical interest in the application of immunotherapeutic strategies to improve patient outcomes in invasive fungal diseases [21–24]. However, such efforts are impaired by the current lack of a high-resolution picture of the fundamental immunology underlying these conditions. Here we provide the first high-parameter, flow cytometric phenotype of peripheral blood leukocytes isolated from CPA patients. Our analysis identified marked neutrophilia in CPA patients, accompanied by a reduction in the frequency of cDC subsets. We observed that the frequency of T-cell memory subsets is maintained in these patients. In our cohort, cDC1s were dysfunctional in their ability to upregulate markers of maturation following *ex vivo* stimulation with the fungal ligand ZYM. Remarkably, we found CPA patients with a history of TB infection had higher frequencies of mature cDC1s than those without, following stimulation. Finally, we demonstrated the potential of a machine learning approach to leverage the immune phenotype of patients to identify CPA patients with poor long-term survival. Together, these data provide an increased depth of understanding of the immune landscape in CPA.

In a previous study, we identified a deficiency in peripheral blood immune cell ability to produce IFNγ when cultured *ex vivo* with fungal PAMPs [3]. However, we did not establish a mechanism responsible for this deficiency. It is therefore striking that here we observed a marked impairment in cDC1s, revealing them to be reduced in frequency in CPA patients’ leukocytes relative to bronchiectasis controls, as has been noted in other respiratory infections including SARS-CoV-2 [25–27] and Influenza A [28], but also impaired in their ability to up-regulate maturation markers and produce IL-12p40, a potent inducer of IFNγ production in T cells, following stimulation with ZYM. The mechanisms by which cDC1s are reduced in frequency in peripheral circulation in CPA patients are unclear. It may be that chronic exposure to pathogen antigens and damage-associated molecular patterns (DAMPs) drives sustained DC recruitment to the site of infection and/or to lung-draining lymph nodes resulting in their sequestration at these sites. However, a study of individuals with SARS-CoV-2 found a reduction of cDC1s in peripheral blood and a near absence of these cells in the BALF [26], whilst another study also observed reduced cDC frequency in the BALF of SARS-CoV-2 patients relative to healthy controls [29], indicating that recruitment to the airways may not explain the loss of circulating cDC1s during infection. However, these data do not exclude recruitment to the lung parenchyma or draining lymph nodes as a mechanism of reduced circulating cDC1 frequency. An alternative explanation for altered distribution is an increase in apoptosis of cDC1s as a consequence of chronic activation [30]. Long-term activation of monocyte-derived DCs in *in vitro* culture resulted in increased signs of apoptosis, reduced capacity to secrete IL-12p70 and an impaired capacity to stimulate IFNγ production in T cells [31]. This reduced capacity to stimulate an IFNγ response mirrors our observations in CPA. Interestingly, we also observed lower Ki67 expression in ZYM-stimulated cDC1s and CD14^+^CD16^-^ monocytes in CPA patients suggesting some reduced capacity for proliferation in response to fungal PAMPs in specific myeloid cell subsets. The mechanism by which this occurs, and whether reduced proliferative capacity contributes to altered myeloid subset frequency in CPA, is unclear and further work is required to validate these observations.

IFNγ is the hallmark cytokine of Th1 cells, the differentiation of which is driven by IL-12-triggered upregulation of the transcription factor T-bet [32]. cDC1s are a key source of IL-12, and their secretion of this cytokine during antigen presentation to T cells is involved in establishing Th1 immunity and driving CD8^+^ T-cell cytotoxicity [33]. It has previously been observed that monocyte-derived DCs, generated from cultured human PBMCs, produce IL-12 and TNFα in response to *in vitro* exposure to *Aspergillus* and can prime Th1 cells [16, 34], indicating that the peripheral, anti-*Aspergillus* DC response should skew towards a type-1 profile under optimal conditions.

Whilst the data presented here does not definitively prove that cDC1 dysfunction is responsible for IFNγ deficiency in CPA patients, it is a promising hypothesis that warrants further investigation. Notably, we did not identify a difference in the T-cell memory phenotype between CPA patients and bronchiectasis controls, supporting the idea that IFNγ deficiency is not a consequence of T-cell dysfunction but rather caused by the failure of cDCs to drive effective Th1 activation. However, deeper analysis of T-cell functionality will help elucidate subtler effects of CPA on the peripheral T-cell compartment not captured in our study. T-cell exhaustion is associated with chronic inflammatory conditions, including infection, and can be a contributing factor in the persistence of disease [35, 36]. While the data presented here do not indicate a role for T cells, specific analyses of markers of T-cell exhaustion in future studies will be essential. Interestingly, in some settings, human blood cDC2s (CD1c^+^ cDCs) have been associated with the ability to prime IFNγ production in an IL-12-independent manner [37]. However, we did not observe high-level IL-12 production by cDC2s in the context of stimulation with fungal-derived ZYM, and it remains unclear whether they contribute to priming IFNγ production in the context of pulmonary aspergillosis via other mechanisms. Notably, in the present study cDC2s showed no deficit in their ability to respond to ZYM when compared to bronchiectasis controls, indicating that impaired cDC2 function is unlikely to be responsible for immune dysfunction in CPA.

In the context of CPA, *Aspergillus* acts as an opportunistic pathogen, typically exploiting a niche created by pre-existing inflammatory conditions. Among the most prevalent of these predisposing conditions is prior TB infection [2, 38]. We observed that CPA patients with a prior history of TB infection had higher frequencies of cDC1s expressing markers of maturation, compared to CPA donors without a reported history of TB, following *ex vivo* ZYM stimulation, an effect that could not be accounted for by difference in ages between the groups. This is striking as TB infection has been associated with a reduced frequency of cDCs in circulation [39, 40] and a reduced capacity of cDCs to induce an IFNγ CD4^+^ T-cell response [41]. However, the way that TB shapes the immune landscape post-resolution of infection is not well understood [42]. Vaccination with bacillus Calmette-Guerin (BCG), live attenuated *Mycobacterium bovis*, has been shown to improve immune responses against pathogens other than *Mycobacterium tuberculosis* [43] via so-called ‘trained immunity’ [44]. Indeed, in mouse models both TB infection and BCG have been shown to be protective against subsequent SARS-CoV-2 challenge [45, 46], with a key role in the priming of an IFNγ response in this process [47]. Further, in a mouse model of BCG-induced protection against lung melanoma metastasis, cDC1 deficient mice lose the protective effect of BCG vaccination [48]. It is tempting to speculate that prior TB infection induces a similar trained response that may enhance the maturation potential of cDC1s when subsequently exposed to fungal PAMPs, supporting their ability to promote a protective IFNγ response. However, given the relatively small sample size available to us, it was not possible to fully account for potential covariates that might explain the observed difference in cDC1 activation potential caused by prior TB infection. Future studies will be essential to delineate the specifics of immune-mediated interaction between TB and CPA, which may be of critical importance given the high co-endemicity of TB and CPA in many parts of the world. Future immunological analyses of CPA should also take BCG vaccination status into account.

In contrast to trained immunity is the occurrence of ‘immune paralysis’ which has been observed in other severe infection settings, most notably sepsis [49]. An impaired, pro-tolerogenic adaptation in peripheral myeloid cells in CPA patients may contribute to the reduced capacity of cDC1s to respond appropriately to ZYM. The changes that lead to immune paralysis are thought to occur at the epigenetic level which were not addressed in this study. Therefore, future studies should investigate whether CPA leads to epigenetic changes in myeloid cells that impair their ability to respond to microbial PAMPs.

A key challenge in the treatment of CPA and many other inflammatory conditions is the ability of clinicians to identify patients with poor survival potential, in order to tailor their therapeutic strategy [2]. Clinical presentation alone may be insufficient to stratify patients into risk groups and there is urgency to develop better methods to meet this need. The rapid development of high-parameter technologies, and the ability to capture large amounts of data on an individual patient basis, has led to a growing interest in the use of machine learning approaches as tools for predicting clinical outcomes and in guiding intervention strategies [50, 51]. We sought to use machine learning to determine whether our immunological parameters could discriminate CPA-infected patients from control bronchiectasis donors. We found that these readouts, in an RF model, were capable of identifying CPA patients from controls with ~70% accuracy. Given that our cohort was relatively small, this is highly encouraging and suggests that the application much larger cohort to the training data would improve the accuracy of machine learning-based models. Remarkably, interrogating the model identified a cluster of patients with a more extreme immune phenotype and a significantly poorer survival probability. These preliminary findings indicate a potential for the application of immune phenotyping-based machine learning in stratifying CPA patients into risk categories. However, we caution that this approach is not suitable to replace standard diagnostic approaches. Instead, we hope that similar approaches developed on significantly larger datasets may be used in parallel to support clinicians in identifying patients with poor survival probabilities that would not otherwise be apparent, thereby allowing for increased monitoring and/or alternative therapeutic approaches. Notably, we only utilized immune parameters in our model. This was in order to establish the utility of these markers on their own terms as the integration of clinical data, particularly those related to features of CPA, would have likely been highly weighted in the model and may have obscured immune-related parameters. However, integrating clinical and demographic information may improve accuracy and should be considered in future studies aiming to refine this approach.

Other than group size, the primary limitation of the present study is that our sampling was restricted to peripheral blood leukocytes. It is well understood that the immune profile of the blood does not always match that in the lungs, with the majority of immune cells (including DCs) present at different frequencies and activation states between sites [52]. Therefore, it is challenging to interpret how directly our peripheral blood data may relate to immunity in the lung during CPA. A study investigating differences in the cytokine response to *Aspergillus* between lung and blood-isolated T cells from donors without CPA found that, whilst blood CD4^+^ T cells preferentially produced IFNγ, lung-derived CD4^+^ T cells skewed strongly towards IL-17 production [53]. This suggests that the local response to *Aspergillus* may be Th17-dominated. Differences in peripheral and local responses to infection have been observed in other disease settings including SARS-CoV-2 [54], influenza [55] and TB [56]. Immune cell function and phenotype are significantly shaped by the local environment through mechanisms such as contact with extracellular matrix components [57], cytokine signalling from tissue-adapted resident immune cells [58], and metabolic adaptation regulated by site-specific metabolite availability [59]. Our previous data indicate that peripheral IFNγ potential is predictive of long-term survival in CPA [3]. Further, our new data demonstrated that peripheral immune phenotype can also discriminate against patients with poor prognosis, suggesting that there is a significant role for peripheral immune readouts providing markers that relate to distinct disease outcomes. One potential explanation for this is that there may be a key role for circulating leukocytes, primed for Th1 functionality, in preventing the progression of infection or invasion of secondary opportunistic pathogens. It will be essential for future studies to also sample the pulmonary environment, to deepen our understanding of which features of the immune response are reflected in the blood or specific to the lung during fungal disease.

Collectively, our data reveal specific aspects of the immune response as major players in CPA, provide additional support for the investigation of immunotherapies as a promising treatment strategy, and demonstrate the potential for immune phenotyping-based machine learning in identifying high-risk patients. Together with our previous results, they provide compelling evidence that peripheral Th1 responses are a key feature of CPA and that cDC1 dysfunction and reduced IL-12 production may explain the impaired IFNγ production that is evident in more severe disease. Thus, our work extends a fundamental understanding of immunity in pulmonary aspergilloses.

## Materials and methods

### Study design

The present study aimed to identify features of the peripheral immune compartment that were associated with CPA. We chose to use patients with bronchiectasis as controls as bronchiectasis is a frequent comorbidity of CPA and is associated with microbially driven airway inflammation; including fungal sensitization. Therefore, a comparison between CPA and bronchiectasis would allow us to identify immune features specific to CPA, as opposed to features common to microbially driven airway disease.

Peripheral blood samples were obtained via the Manchester Allergy, Respiratory and Thoracic Surgery (ManARTs) biobank. CPA patients were recruited from the National Aspergillosis Centre clinic and control bronchiectasis patients were recruited from the North West Lung Centre clinic, both in the Manchester University NHS Foundation trust. Written consent was obtained from all donors and the study used existing biobank ethics approval (Ref 15/NW/0409) in accordance with the standards outlined in the Helsinki Declaration.

A total of 25 CPA donors and 25 control bronchiectasis donors were recruited. CPA was diagnosed based on consensus criteria [60]. Two CPA donors were excluded from the final analysis due to a subsequent change in diagnosis. Two bronchiectasis donors were not included in the analysis as one withdrew consent following donation and one did not provide a sufficient blood sample for cell isolation. In some cases, due to limited recovered cell numbers, it was not possible to run all immune phenotyping panels for every donor (Supplementary Table S1).

### Sex a biological variable

For both groups, both male and female donors were recruited (Table 1). Based on our previous work [3], we did not anticipate sex-based differences in the immune phenotype of CPA. However, between-sex statistical comparisons were performed on the data presented here (Fig. 4A, Supplementary Fig. S3B).

### Cell isolation, storage, and stimulation

Peripheral blood was isolated into EDTA-coated tubes from donors and transported to the University of Manchester at ambient temperature. One milliliter of blood was used to isolate all circulating leukocytes by RBC lysis. 6 ml of blood was used to isolate PBMCs.

For isolation of total leukocytes by RBC lysis, 1 ml of blood was diluted in 8 ml of sterile dH_2_O and incubated at room temperature for 3 min. Immediately after incubation 1 ml of 10X PBS (Sigma–Aldrich) was added to the diluted sample. Leukocytes were pelleted by centrifugation (500 xg for 5 min) and resuspended in 1 ml of PBS (Sigma–Aldrich) for counting. The cells were then pelleted by centrifugation (500 xg for 5 min) and resuspended in freezing media (10% DMSO (Sigma) in foetal calf serum (FCS (Sigma))) at 1–3 × 10^6^ cells/ml. Cells were frozen to −80^°^C at a rate of −1^o^C/min then transferred to −150^o^C for storage prior to analysis.

For isolation of PBMCs whole blood was diluted at a 1:1 ratio with PBS. The sample was transferred to a 50 ml LeucoSep tube (Greiner), containing 15 ml of Ficoll Paque (Cytiva), and centrifuged at 800 xg for 15 min with the brake off. The PBMC fraction was transferred to a fresh tube. Cells were pelleted by centrifugation (500 xg for 5 min) and washed with 10 ml of PBS. Cells were then counted, frozen, and stored as described above.

Cells were thawed by incubating in a 37°C water bath. Once thawed cells were immediately diluted 1:10 in pre-warmed culture media (RPMI1640 (Sigma-Aldrich) + 10% FCS + 2 mM l-glutamine (Sigma–Aldrich) + 100 U/ml penicillin + 100 μg/ml streptomycin (Sigma–Aldrich)). Cells were pelleted by centrifugation (500 xg for 5 min) and washed in culture media.

For stimulation cells were seeded in a sterile u-bottom 96 well plate (Thermo Scientific) (1-2 × 10^6^ cells/well) and rested overnight in a 37^o^C (5% CO_2_) incubator in 200 μL of culture media. Cells were stimulated with Zymosan (ZYM) (InvivoGen) (10 μg/ml) in culture media in the presence of Protein Transport Inhibitor Cocktail for 16 hrs (eBioscience).

### Flow cytometry

Cells were transferred to a v-bottom 96 well plate (Thermo Scientific). Cells were washed twice with PBS and then incubated with Zombie UV dye (BioLegend) (1:2000) for 15 min at room temperature. Cells were then washed with flow buffer (PBS + 1% FCS + 2 mM EDTA (Sigma-Aldrich)) and blocked with Human BD FcBlock (BD Parmingen) at 4^°^C for 10 min. Cells were then incubated with surface antibodies, diluted in 35 μL of flow buffer, for 40 min at 4^°^C (Supplementary Table S2). For panels that required intracellular staining, cells were then fixed and permeabilized using the eBioscience Foxp3/Transcription Factor Fixation/Permeabilization kit as directed by the manufacturer. Cells were incubated overnight at 4^o^C with intracellular antibodies then washed and resuspended in flow buffer. Data was acquired on a BD LSRFortessa Cell Analyzer and analysed using FlowJo (v10.10.0).

### Statistical analysis

Statistical analysis was performed using R (R core team) and figures were produced using the ‘ggplot2’ package. Statistical comparisons were performed using the ‘rstatix’ package. All statistical values were calculated using unpaired Wilcoxon two-sample tests. Where appropriate *P* value adjustment to correct for multiple-testing was performed using the Holm method, this is noted in the figure legends. A *P* value < 0.05 was considered statistically significant.

Random Forests analysis was performed using the ‘randomForest’ package. Missing data-points were imputed using the ‘rfImpute’ function. The number of trees was set at 1000 and the mtry parameter was set at 3 after optimisation to identify the settings that produced the lowest out-of-bag error rate. Classical multi-dimensional scaling (MDS) was performed by generating a distance matrix from the Random Forests model using the ‘dist’ function and then running the ‘cmdscale’ function.

Kaplan-Meier survival analysis was performed with the ‘survival’ and ‘survminer’ packages. Statistical comparison between Kaplan-Meier curves was performed using a log-rank test.

## Supplementary Material

uxaf038_suppl_Supplementary_Figures_S1-S4_Tables_S1-S2

## Data Availability

Values for all data points in graphs are reported in the Supporting Data Values file.

## Abbreviations

CCR7: C-C chemokine receptor type 7
cDC1: conventional type-1 dendritic cell
cDC2: conventional type-2 dendritic cell
COPD: chronic obstructive pulmonary disease
CPA: chronic pulmonary aspergillosis
DC: dendritic cell
IFNγ: interferon gamma
IL: interleukin
MNP: mononuclear phagocyte
NTM: non-tuberculosis mycobacterial infection
PAMP: pathogen-associated molecular pattern
pDC: plasmacytoid dendritic cell
RF: random forests
TB: tuberculosis
Th: T helper
TLR: toll-like receptor
TNAα: tumour necrosis factor alpha
ZYM: Zymosan
