# Supplementary material for: Dendritic cell dysfunction, including impaired IL-12 production, is associated with chronic pulmonary aspergillosis
Source: Clin Exp Immunol. 2025 Jun 12;219(1):uxaf038. doi: 10.1093/cei/uxaf038 (PMC12231563; doi:10.1093/cei/uxaf038)
Supplement: uxaf038_suppl_Supplementary_Figures_S1-S4_Tables_S1-S2 [file uxaf038_suppl_supplementary_figures_s1-s4_tables_s1-s2.docx]

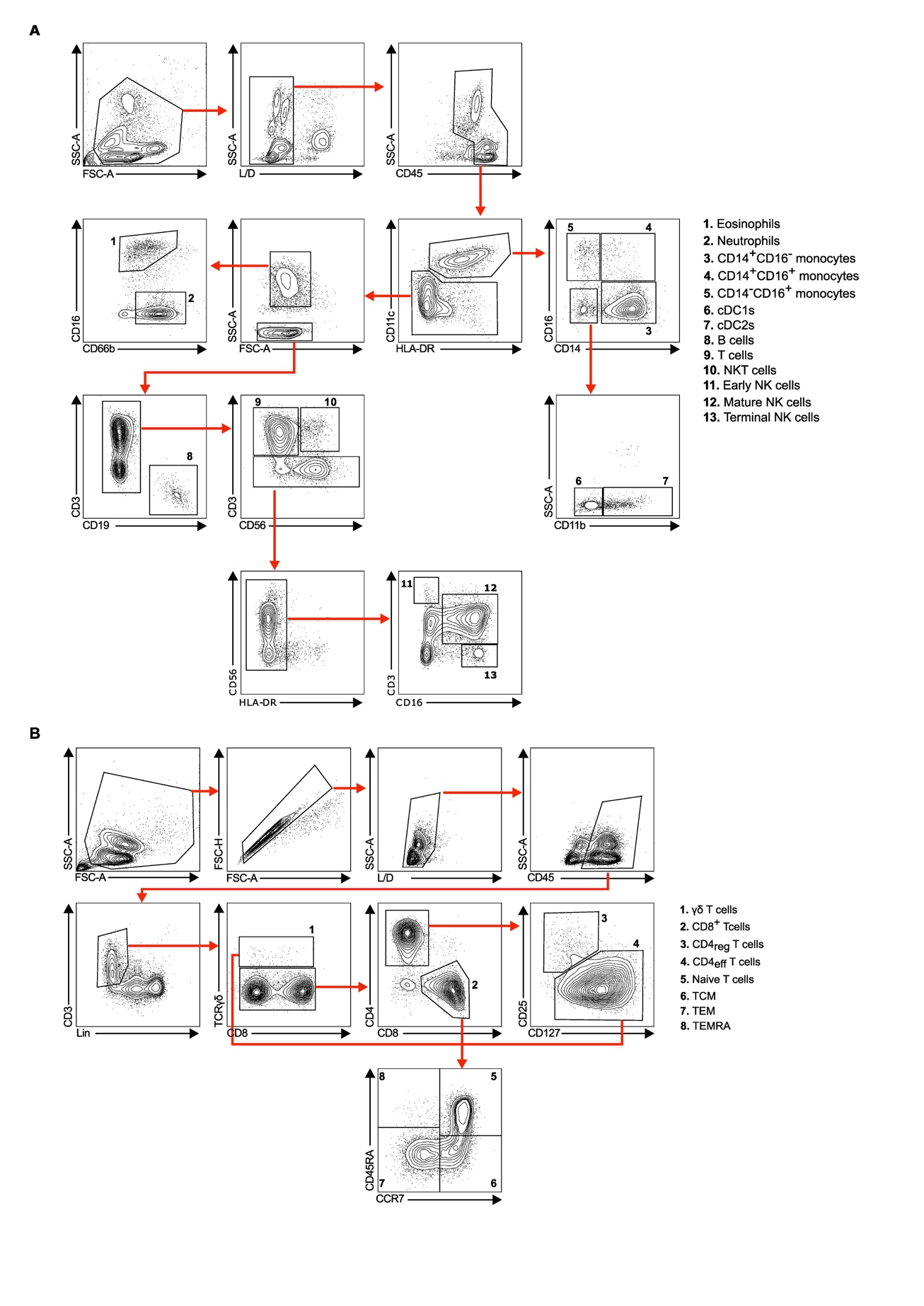


**Fig. S1. Representative gating strategies.** Flow cytometric gating strategies for identification of different immune cell subsets. (A) Strategy for identification of immune cell subsets in total leukocytes isolated by RBC lysis. (B) Strategy for identification of T cell subsets in PBMCs.


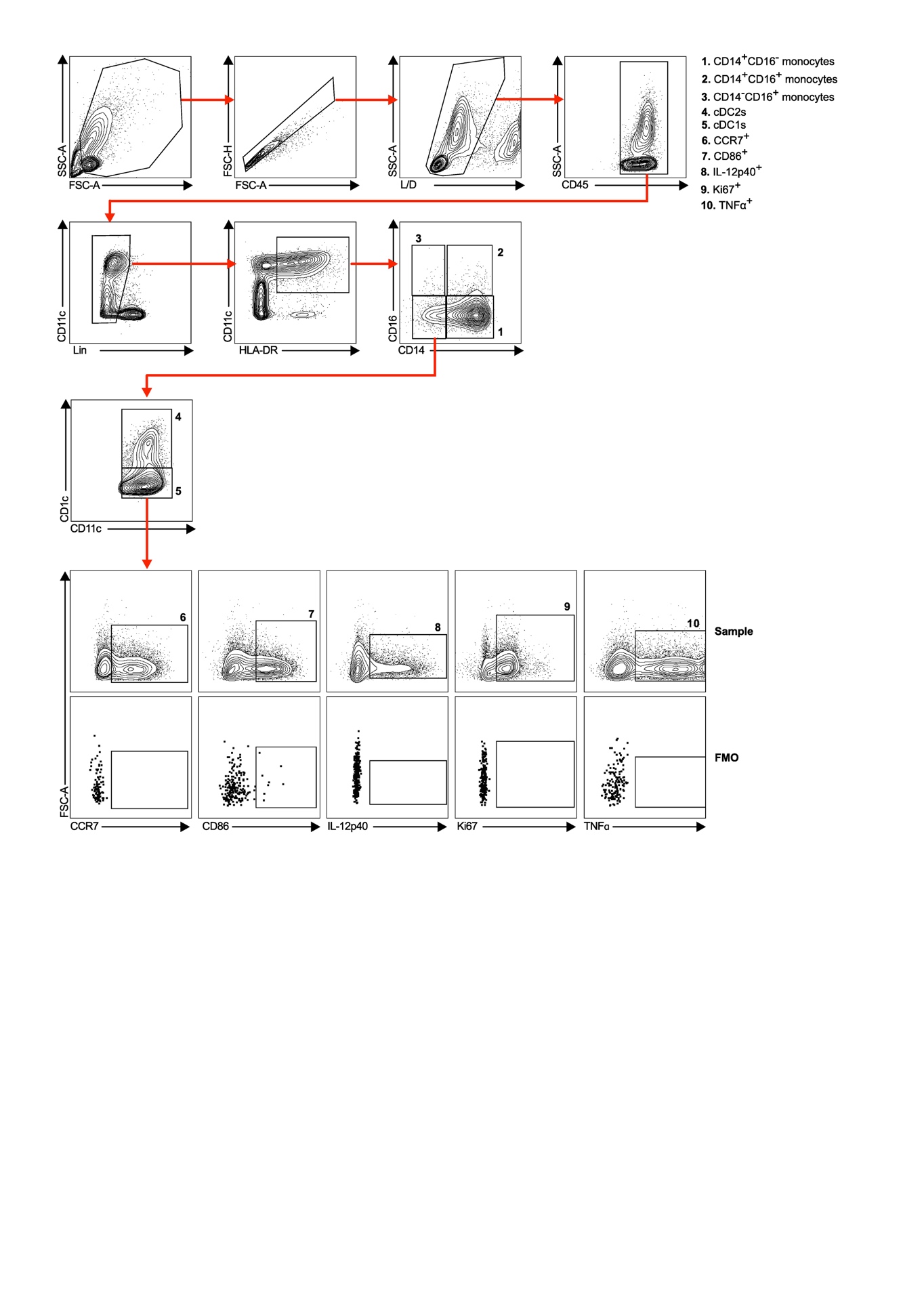


**Fig. S2. Representative gating strategy.** Flow cytometric gating strategy for the identification of monocyte and dendritic cell subsets and the expression of phenotypic markers.


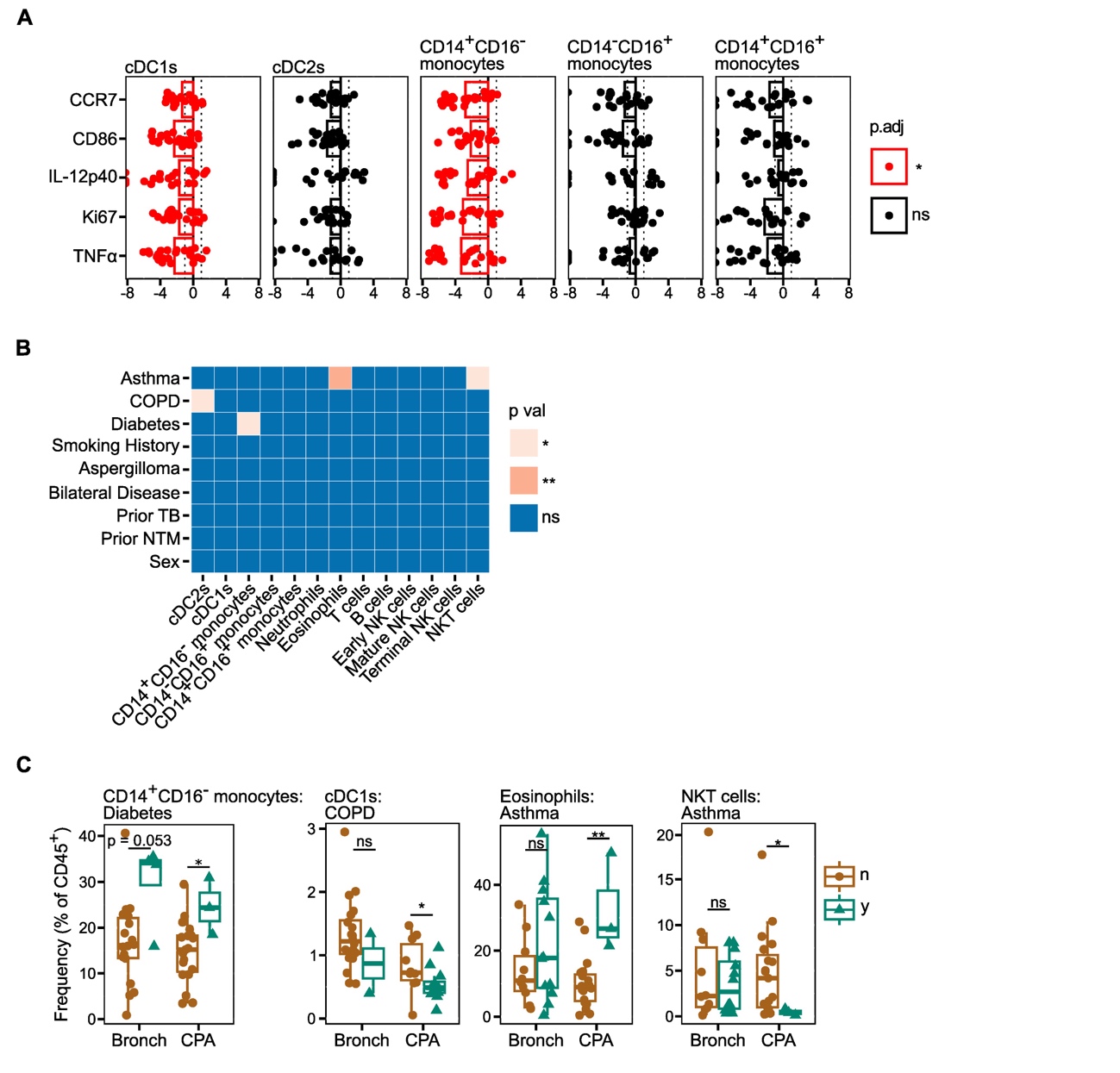


**Fig. S3. Frequency of activation marker expressing myeloid cells.** (**A**) Scatter plots indicating the log_2_(fold change) of the frequency of marker positive cells, as a proportion of total CD45^+^ cells, in CPA donors relative to the median of the bronchiectasis (Bronch) control group. Points represent individual donors, bars indicate the mean log_2_(fold change) of the CPA group, dotted lines indicate -1 and 1, colour denotes the adjusted p value (p.adj) for statistical comparison between the CPA group and control group as calculated by unpaired Wilcoxon test with correction using the Holm correction method. (**B**) Tile plot indicating the results of statistical comparisons for CPA donors based on clinical covariates, in which the frequency of immune cell subsets as a proportion of total CD45^+^ cells (as identified in Fig. 1) is compared between donors with that covariate (y) compared to those without (n), e.g. CPA donors with Asthma (y) compared to those without asthma (n). Each tile indicates a statistical comparison. Tile colour indicates the calculated p value as calculated by unpaired Wilcoxon test (**C)** Boxplots indicating the frequency of immune cell subsets in CPA and Bronch donors stratified by clinical co-variates. Boxplots indicate the median and interquartile ranges. Whiskers indicate the minimum/maximum value within 1.5x the lower/upper quartile limit. Points indicate individual donors. * p < 0.05, ** p < 0.01.


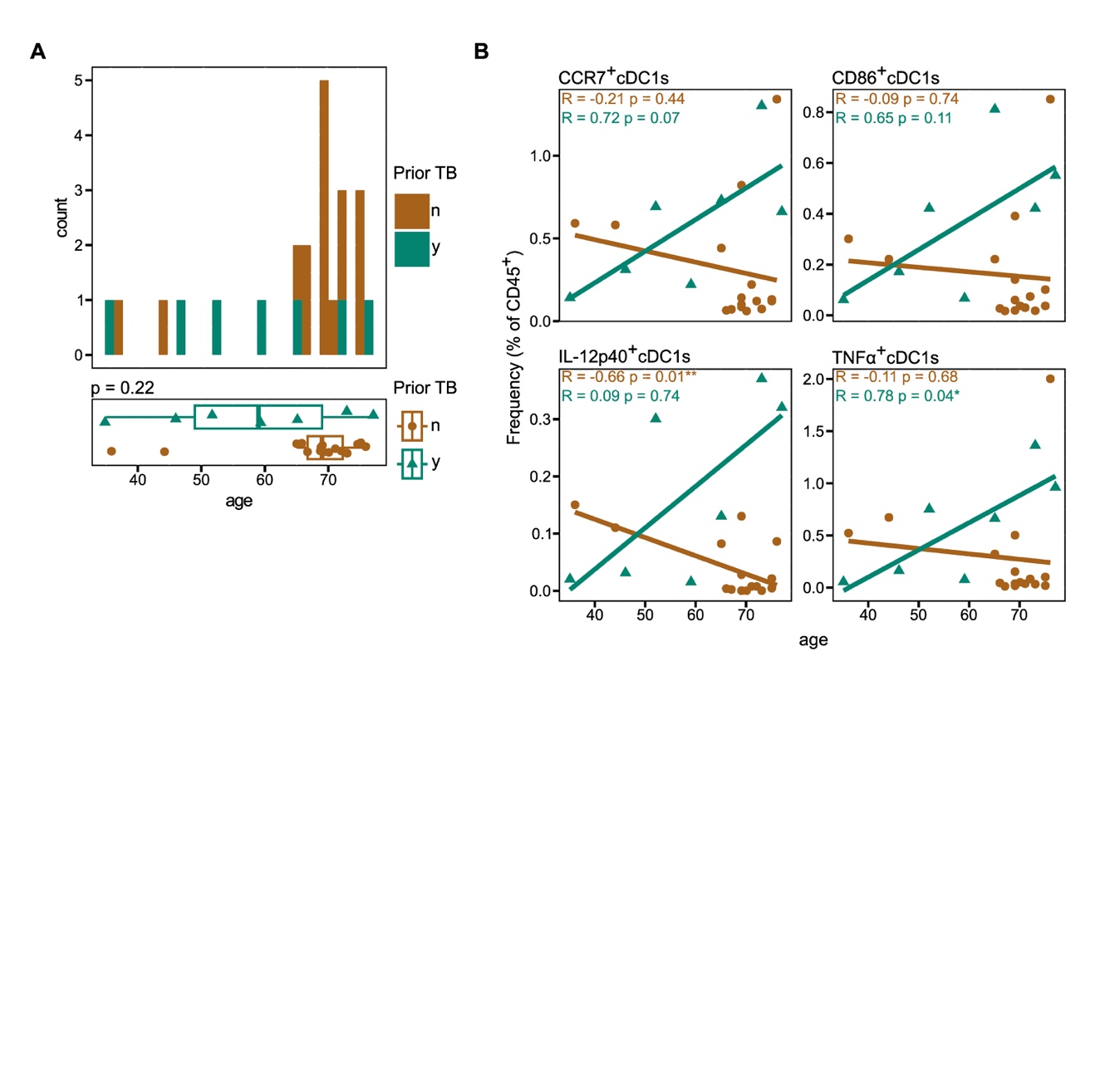


**Fig. S4. The relationship between age and cDC1 activation marker expression in CPA patients stratified by prior TB status.** (**A**) Histogram and boxplot showing the age distribution within the CPA group stratified by prior TB infection. n = no prior TB infection, y = prior TB infection. P value was calculated by unpaired Wilcoxon test. (**B**) Scatter plots correlating age with the frequency of marker^+^ cDC1s as a proportion of total CD45^+^ cells following *ex vivo* stimulation with Zymosan. CPA patients were stratified by prior TB infection. R and p values were calculated by Pearson’s correlation test. Lines represent the fitted linear regression.

**Table S1. Summary of data acquisition for each donor**

| **Donor** | **group** | **Panel 1 – broad immune phenotyping** | **Panel 2 – T cell phenotyping** | **Panel 3 – post ZYM myeloid activation** |
| --- | --- | --- | --- | --- |
|  |  | Fig 1, Fig 5 | Fig 2 | Fig 3, Fig 4, Fig 5 |
| Donor 1 | CPA | Y | Y | Y |
| Donor 2 | CPA | Y | N | Y |
| Donor 3 | CPA | Y | Y | Y |
| Donor 4 | CPA | Y | Y | Y |
| Donor 5 | Bronch | Y | Y | Y |
| Donor 6 | CPA | Y | Y | Y |
| Donor 7 | CPA | Y | Y | Y |
| Donor 8 | CPA | Y | N | Y |
| Donor 9 | Bronch | Y | N | N |
| Donor 10 | Bronch | Y | Y | Y |
| Donor 11 | Bronch | Y | N | Y |
| Donor 12 | Bronch | Y | N | Y |
| Donor 13 | Bronch | Y | N | Y |
| Donor 14 | CPA | Y | N | Y |
| Donor 15 | CPA | Y | Y | Y |
| Donor 16 | CPA | Y | N | Y |
| Donor 17 | Bronch | Y | Y | Y |
| Donor 18 | CPA | Y | Y | Y |
| Donor 19 | Bronch | Y | Y | Y |
| Donor 20 | CPA | Y | Y | Y |
| Donor 21 | Bronch | Y | Y | Y |
| Donor 22 | Bronch | Y | Y | Y |
| Donor 23 | Bronch | Y | Y | Y |
| Donor 24 | Bronch | Y | Y | Y |
| Donor 25 | Bronch | Y | Y | Y |
| Donor 26 | Bronch | Y | Y | Y |
| Donor 27 | Bronch | Y | Y | Y |
| Donor 28 | Bronch | Y | Y | N |
| Donor 29 | CPA | Y | Y | Y |
| Donor 30 | Bronch | Y | Y | Y |
| Donor 31 | Bronch | Y | Y | Y |
| Donor 32 | Bronch | Y | N | Y |
| Donor 33 | Bronch | Y | Y | Y |
| Donor 34 | CPA | Y | Y | Y |
| Donor 35 | Bronch | Y | Y | Y |
| Donor 36 | Bronch | Y | Y | Y |
| Donor 37 | CPA | Y | Y | Y |
| Donor 38 | CPA | Y | N | Y |
| Donor 39 | CPA | Y | N | Y |
| Donor 40 | CPA | Y | Y | Y |
| Donor 41 | CPA | Y | N | Y |
| Donor 42 | CPA | Y | Y | Y |
| Donor 43 | CPA | Y | Y | Y |
| Donor 44 | CPA | Y | Y | Y |
| Donor 45 | CPA | N | Y | Y |
| Donor 46 | Bronch | N | N | Y |

Table denotes which donors were included in each immune phenotyping flow cytometry panel. Y = data was acquired for a given patient, N = data was not acquired for a given patient.

**Table S2. Antibodies used for flow cytometry**

| **Marker** | **Fluorophore** | **Dilution** | **Clone** | **Manufacturer** |
| --- | --- | --- | --- | --- |
| **Panel 1** | | | |  |
| CD56 | FITC | 1:50 | 5.1H11 | BioLegend |
| CD27 | PerCP-Cy5.5 | 1:50 | M-T271 | BioLegend |
| CD66b | AF700 | 1:200 | G10F5 | BioLegend |
| CD3 | APC-ef780 | 1:200 | OKT3 | Invitrogen |
| IgD | BV421 | 1:50 | IA6-2 | BioLegend |
| CD45 | BV510 | 1:200 | 2D1 | BioLegend |
| CD11c | BV605 | 1:50 | B-ly6 | BD Biosciences |
| HLA-DR | BV711 | 1:150 | L243 | BioLegend |
| CD16 | BV785 | 1:200 | 3G8 | BioLegend |
| CD14 | PE | 1:200 | 63D3 | BioLegend |
| CD11b | PE-CF594 | 1:200 | ICRF44 | BD Biosciences |
| CD19 | PE-Cy5 | 1:200 | H1B19 | BioLegend |
| CD64 | PE-Cy7 | 1:200 | 10.1 | BioLegend |
| **Panel 2** | | | |  |
| Lin (CD56, CD19, CD14) | FITC | 1:200, 1:200, 1:200 | 5.1H11, H1B19, 63D3 | BioLegend |
| CD8a | PerCP-ef710 | 1:200 | SK1 | Invitrogen |
| CD4 | AF700 | 1:200 | SK3 | BioLegend |
| CD3 | APC-ef780 | 1:200 | OKT3 | Invitrogen |
| CD45 | BV510 | 1:200 | 2D1 | BioLegend |
| CD45RA | BV605 | 1:200 | HI100 | BioLegend |
| TCRγδ-biotin, SA-BV650 | BV650 | 1:100, 1:200 | B1 | BioLegend |
| CD127 | BV711 | 1:100 | A019D5 | BioLegend |
| CD25 | BV785 | 1:50 | BC96 | BioLegend |
| CCR7 | PE-Cy7 | 1:150 | G043H7 | BioLegend |
| **Panel 3** | | | |  |
| Lin (CD56, CD19, CD3, CD66b) | FITC | 1:200, 1:200, 1:200, 1:200 | 5.1H11, H1B19, OKT3, G10F5 | BioLegend |
| CD11b | PerCP-Cy5.5 | 1:200 | ICRF44 | BioLegend |
| CD80 | APC | 1:200 | 2D10 | BioLegend |
| HLA-DR | AF700 | 1:300 | LN3 | BioLegend |
| CD14 | APC-ef780 | 1:300 | 61D3 | Invitrogen |
| CD1c | BV421 | 1:200 | L161 | BioLegend |
| CD45 | BV510 | 1:400 | 2D1 | BioLegend |
| TNFα (IC) | BV605 | 1:400 | Mab11 | BioLegend |
| CD86 | BV650 | 1:200 | IT2.2 | BioLegend |
| CD16 | BV711 | 1:300 | 3G8 | BioLegend |
| IL-12/IL-23p40 (IC) | PE | 1:200 | C11.5 | BioLegend |
| Ki-67 (IC) | PE-CF594 | 1:200 | Ki-67 | BioLegend |
| CD11c | PE-Cy5 | 1:200 | B-ly6 | BD Biosciences |
| CCR7 | PE-Cy7 | 1:150 | G043H7 | BioLegend |
